# Quality prediction of synthesized speech based on tensor structured EEG signals

**DOI:** 10.1371/journal.pone.0193521

**Published:** 2018-06-14

**Authors:** Hayato Maki, Sakriani Sakti, Hiroki Tanaka, Satoshi Nakamura

**Affiliations:** Graduate School of Information Sciences, Nara Institue of Science and Technology, Ikoma, Nara, Japan; Boston Children’s Hospital / Harvard Medical School, UNITED STATES

## Abstract

This study investigates quality prediction methods for synthesized speech using EEG. Training a predictive model using EEG is challenging due to a small number of training trials, a low signal-to-noise ratio, and a high correlation among independent variables. When a predictive model is trained with a machine learning algorithm, the features extracted from multi-channel EEG signals are usually organized as a vector and their structures are ignored even though they are highly structured signals. This study predicts the subjective rating scores of synthesized speeches, including their overall impression, valence, and arousal, by creating tensor structured features instead of vectorized ones to exploit the structure of the features. We extracted various features to construct a tensor feature that maintained their structure. Vectorized and tensorial features were used to predict the rating scales, and the experimental result showed that prediction with tensorial features achieved the better predictive performance. Among the features, the alpha and beta bands are particularly more effective for predictions than other features, which agrees with previous neurophysiological studies.

## Introduction

Text-to-Speech (TTS) systems, which convert a written text into speech, and are becoming more widely implemented in mobile phones, car navigation systems, and other consumer electronics. Such systems play a critical role in many applications because speech is the most fundamental and easiest communication tool for human beings. Therefore, synthesized speeches must sound natural for good machine-to-human communications.

The research of TTS systems needs reasonable criteria that evaluate the qualities of synthesized speeches. Several current evaluation methods have their own advantages and disadvantages: (1) subjective ratings [1–3], (2) analyzing a speech signal itself [4–6], and (3) measuring the physiological responses of listeners to speech [7–14].

In the first approach, the two most common aspects for quality judgment are naturalness and intelligibility. Naturalness describes how close synthesized speech is to human speech, and intelligibility reflects how well the speech content can be heard. The former is usually measured by a mean opinion score (MOS) test [1], and the latter is gauged by semantically unpredictable sentences (SUS) [3]. In addition, valence and arousal are often used to evaluate the subjective impressions of speech [11, 13, 15, 16] and to model emotions [17–20]. Valence reflects a positive or a negative emotion. Arousal reflects the degree of intensity or activation. In a MOS test, subjects listen to speech and rate its relative perceived quality on some kind of a scale, for example, “excellent,” “good,” “fair,” “poor,” “bad.” Then the scores are averaged across subjects. This is well established method for which references on how to perform it are available [2], making it the only standard way to evaluate the naturalness quality of synthesized speech. However, their appropriateness has not been fully proven because high inter- and intra-subject inconsistencies are often observed in the ratings, resulting in poor reproductivity [21].

In the second approach, speech quality is automatically evaluated at its signal level by software that inputs a speech file and outputs the estimated speech quality. Advantages of these methods include complete reproductivity and less time consumption after such software is developed. However, appropriateness is difficult to prove because the exact relationship between the acoustic features and the perceived quality of speech by a listener is not well understood [21]. In fact, speech quality must be evaluated not only physically but also psychologically because it is commonly defined as an assessment result within which a listener compares his/her perceptions with expectations [22, 23].

Last, quality estimation methods are emerging that measure the physiological responses of a listener [24]. Even though these methods have not been established yet, they are worth investigating because physiological signals can be recorded automatically and continuously to provide insight about listener’s cognitive states without interruptions caused by directly asking him/her to answer questions. Among existing non-invasive physiological response measures, electroencephalography (EEG) has especially great potential to estimate a listener’s perceived speech qualities for the following reasons. EEGs can be recorded at a higher temporal resolution, e.g., a millisecond range, than hemodynamic measures, including functional magnetic resonance imaging (fMRI) and functional near-infrared spectroscopy (fNIRS), both of which analyze the changes in blood flow that inherently take a few seconds until a brain response can be recorded. Temporal resolution is important to evaluate speech quality since the temporal structure of speech largely affects its perceived quality. In addition, an EEG recording equipment is relatively small, less expensive than other brain recording equipments, and can be even wireless, which allows us to use it in daily environments, whereas fMRI and magnetoencephalography (MEG) can only be used in experimental rooms because of the lack of portabity. Measuring physiological responses to speech in daily environments is critical because speech is everywhere. Despite the above advantages, the main disadvantage of physiological measures is the difficulty of data gathering. The amount of data that can be collected from a subject is limited for practical and ethical reasons. Conducting experiments is usually time-consuming and labor-intensive. In addition, physiological data are generally noisy and easily contaminated by artifacts. Furthermore, multi-channel EEG signals are usually highly correlated to each other, which makes the features extracted from them less informative compared to the height of their dimensions. These aspects of EEG (limited amount of data, noise, and high correlation and dimension) complicate training a predictive model with EEG data and require a sophisticated dimension reduction or regularization techniques [25].

Existing researches have analyzed EEG responses to speech stimuli using event-related potentials (ERP), which are time-locked responses to external or internal events in terms of a voltage change that are usually visualized and quantified after synchronous averaging of multiple epochs [7–9]. Due to its definition, measuring ERP need the instantaneous time-locking points at which an event occurs, complicating the use of ERP if stimuli onsets are gradual or unclear [26]. Therefore, ERP is not suitable for our purpose of the predicting perceived quality of speech whose length exceeds a second because it is usually unclear which time points affect a listener’s perceived quality. Other research used power spectral density [14, 27] and their difference between EEG channels [11, 13] at multiple frequency bands. Neuroscience studies reported that EEG spectral changes in distinct regions and between hemispheres are related to emotions [28–31]. Other studies used EEG phase synchronization between EEG channel pairs and found a correlation to emotions [32, 33].

The purpose of this research is to predict the perceived qualities of synthesized speeches using only EEG. Interest is growing in the development of a machine learning algorithm that uses an input/output data structure as tensor formats [34–36]. Such tensor structured features were investigated in this study because EEG signals can have structures in time, frequency, space, experimental condition, and other modalities.

## Materials

We used the PhySyQX data set [10], which consists of speech files, their subjective rating scores from 21 subjects, and EEG signals from the same subjects recorded while they listened to the speech. The data recording protocol was approved by the INRS Research Ethics Office, and participants gave informed consent for their participation and to make their data anonymous and freely available online. The details of the data set and the experimental procedures are available in [10]. We obtained it by an e-mail request.

### Speech stimuli

The speech stimuli presented to the subjects in the data set consist of speech collected from four humans and seven commercially available TTS systems. From each human and each TTS system, four English sentences were collected, whose durations ranged from 13 to 22 seconds. The 44 human and synthesized speeches were presented to each subject in random order.

### Experimental procedure

**Fig 1:**
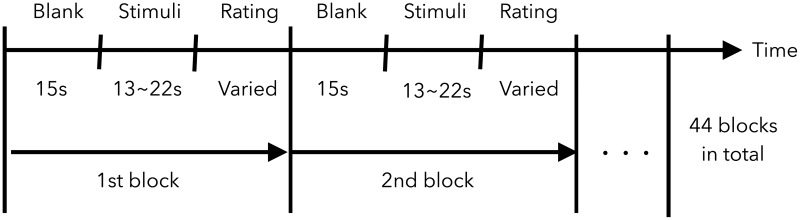
Timeline of EEG and subjective evaluation data recording experiment.

The experiment’s timeline is shown in Fig 1. A 15-second rest period was provided before each stimulus presentation. It is followed by a subjective rating period during which the subjects evaluated the speech to which they had just listened. The subjective rating scales used in this study are shown in Table 1 and include overall impression (MOS), valence (VAL), and arousal (ARL). MOS was evaluated with a 5-scale rating and the others with a 9-scale using self-assessment manikin [37].

**Table 1:** Subjective rating scales.

Rating Scale	Abbreviation	Description
Overall Impression	MOS	1 (Bad) to 5 (Excellent)
Valence	VAL	1 (Negative) to 9(Positive)
Arousal	ARL	1 (Unexcited) to 9 (Excited)

#### EEG recording and preprocess

EEG data were recorded throughout the experiment with 64 scalp channels. The sampling rate was 512 Hz, which was down-sampled to 256 Hz. All the channels were placed on scalp according to the modified 10/20 system [38]. Some channels were removed from further analysis because they were noisy. A band-pass filter was applied to all the data between 0.5–50 Hz and applied an independent component analysis based semi-artifact removal technique using the ADJUST toolbox [39]. After these preprocessing, the EEG signal of each subject was cut into 44 epochs corresponding to the stimuli listening periods.

## Methods

### Feature extraction

All features were extracted at five frequency bands from a channel or a channel pair. The frequency bands include delta (*δ*: 1–4 Hz), theta (*θ*: 4–8 Hz), alpha (*α*: 8–12 Hz), beta (*β*: 12–30 Hz), and gamma (*γ*: 30–45 Hz). Let us denote the Fourier transformation at the frequency of *f*_*k*_ of the *n*-th trial recorded by the *m*-th channel by ***x***_*n*,*m*_(*f*_*k*_). An estimator of the power spectrum density and a phase spectrum denoted by *p*_*k*_ and *h*_*k*_ can be calculated using the periodogram method as follows:

$$\begin{array}{c} p_{n,m}\left(f_{k}\right)=\frac{1}{T}{\left|x_{n,m}\left(f_{k}\right)\right|}^{2} \end{array}$$ (1)

$$\begin{array}{c} h_{n,m}\left(f_{k}\right)=\text{angle}\left(x_{n,m}\left(f_{k}\right)\right), \end{array}$$ (2)

where *T* is the number of time samples within a trial. Then, we averaged the power spectrum density over the frequency bins within the range of each frequency band to define channel-based features PSD_*n*_(*m*, *f*) as follows:

$$\begin{array}{c} {\text{PSD}}_{n}\left(m,f\right)=\frac{1}{|D_{f}|}\sum_{f_{k}\in D_{f}}p_{n,m}\left(f_{k}\right), \end{array}$$ (3)

where *D*_*f*_ is the index set of the frequency bins included in the range of the *f*-th frequency band and |*D*_*f*_| is the number of the elements in *D*_*f*_. The channel-pair-based features are also defined using the averaged power spectrum density and the phase spectrum as follows:

$$\begin{array}{c} {\text{PWD}}_{n}\left(m_{1},m_{2},f\right)=\text{PSD}\left(m_{1},f\right)-{\text{PSD}}_{n}\left(m_{2},f\right) \end{array}$$ (4)

$$\begin{array}{c} {\text{PHD}}_{n}\left(m_{1},m_{2},f\right)=\frac{1}{|D_{f}|}\sum_{f_{k}\in D_{f}}h_{n,m_{1}}\left(f_{k}\right)-h_{n,m_{2}}\left(f_{k}\right). \end{array}$$ (5)

If *M* EEG channels and *F* frequency bands are used (*F* = 5 in this study), *I* = *F*(*M*(*M* − 1) + *M*) features are calculated. The feature matrix X can be expressed as:

$$\begin{array}{c} X={\left(\begin{array}{c} x(1),x(2),\ldots ,x(N) \end{array}\right)}^{⊤}\in R^{N\times I}, \end{array}$$ (6)

where *N* is the number of training trials and ***x***(*n*) is a feature vector of the *n*-th trial and has all the features PSD_*n*_, PWD_*n*_, and PHD_*n*_.

To exploit structures of the features, we organized the features as a tensor $X\in R^{N\times M\times M\times F}$ as follows:

$$\begin{array}{c} X\left(n,m_{1},\;m_{2},f\right)=\{\begin{array}{cc} {\text{PWD}}_{n}\left(m_{1},m_{2},f\right) & \left(m_{1}>m_{2}\right)\\ {\text{PHD}}_{n}\left(m_{1},\;m_{2},f\right) & \left(m_{1}<m_{2}\right)\\ {\text{PSD}}_{n}\left(m_{1},f\right) & \left(m_{1}=m_{2}\right) \end{array}. \end{array}$$ (7)

**Fig 2:**
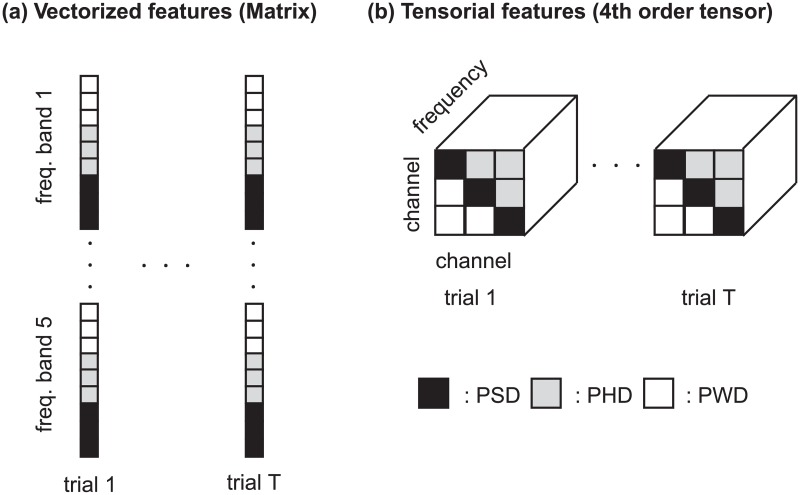
Schematic image of vectorized and tensorial features. (a) Vectorized feature is included in a matrix whose first and second modes are trials and features. PWD, PHD, and PSD at each frequency band are lined as a vector in each trial. (b) Tensor structured dependent variable with four modes: trials, channel-1, channel-2, and frequency bands. Tensor elements with a larger channel-1 index than channel-2 are PWD, and a smaller channel-1 index than channel-2 are PHD, and identical channel indexes are PSD.

The feature matrix and tensor are depicted in Fig 2.

### Regression analysis

Higher order partial least square (HOPLS) [34] and standard partial least square (PLS) [40, 41] simultaneously perform dimension reduction and regression, which were used in this study. The former is a natural extension of the latter so that tensor-format features can be used.

Let us denote the response matrix by Y that has all the response variables of all training trials:

$$Y={\left(y(1),y(2),\ldots ,y(N)\right)}^{⊤}\in R^{N\times J},$$ (8)

where ***y***(*n*) is the *J* = 3 dimensional response vector of the *n*-th trial. All response variables were normalized to have zero mean and unit variance.

PLS performs a simultaneous decomposition of X and Y to find common latent variables $t_{r}\in R^{N}$ as:

$$X=\sum_{r=1}^{R_{1}}t_{r}p_{r}^{⊤}+E$$ (9)

$$Y=\sum_{r=1}^{R_{1}}t_{r}q_{r}^{⊤}+F,$$ (10)

where *E* and *F* are the residual matrices, and *R*_1_ is called the number of the components.

On the other hand, HOPLS can be similarly formulated as the problem to find latent variables as follows:

$$\begin{array}{c} X=\sum_{r=1}^{R_{2}}G_{r}\times_{1}t_{r}\times_{2}P_{r}^{\left(1\right)}\times_{3}P_{r}^{\left(2\right)}\times_{4}P_{r}^{\left(3\right)}+E \end{array}$$ (11)

$$\begin{array}{c} G_{r}\in R^{1\times M\times M\times F},\;P_{r}^{\left(k\right)}\in \{\begin{array}{cc} R^{M\times L_{k}} & \left(k=1,2\right)\\ R^{F\times L_{k}} & \left(k=3\right) \end{array} \end{array}$$ (12)

$$\begin{array}{c} Y=\sum_{r=1}^{R_{2}}t_{r}q_{r}^{⊤}+V, \end{array}$$ (13)

where $G_{r}$ is called the core tensor, E and V are the residuals, *R*_2_ is the number of the components, and ×_*k*_ denotes the *k*-mode product [42]. $P_{r}^{\left(n\right)}$ is called the loading matrix of the *r*-th component, and *L*_*k*_ is called the number of the *k*-mode loadings.

If data are plentiful, which is rare in EEG studies, the best approach for training and evaluating the performance of a predictive model is to randomly divide the dataset into three parts: training, validation, and test sets, which are respectively used to train a model, tune hyper-parameters or select a model, and evaluate the generalization error [43]. However, since the amount of data in this study is too small to exploit such an ideal protocol, we instead used leave-one-out cross-validation for each subject. The hyper-parameter *R*_1_ of PLS varied from 1 to 43, loadings of the channel-1 *L*_1_ and the channel-2 *L*_2_ ranged from 1 to 7. The loading gs of the frequency band *L*_3_ and the number of components *R*_2_ of HOPLS ranged from 1 to 5. The result of the models that achieved the best performance was reported in Results.

### Evaluation metrics

Root mean squared error (RMSE) was used to quantify the predictability of the regression models for each subject, which are formulated as:

$$\begin{array}{c} \text{RMSE}=\sqrt{\frac{1}{N}\sum_{i=1}^{N}{\left({\hat{y}}_{i}-y_{i}\right)}^{2}}, \end{array}$$ (14)

where *N* is the number of test samples, ${\hat{y}}_{i}$ is the predicted value for the *i*-th test data, and *y*_*i*_ is the actual value.

## Results

**Fig 3:**
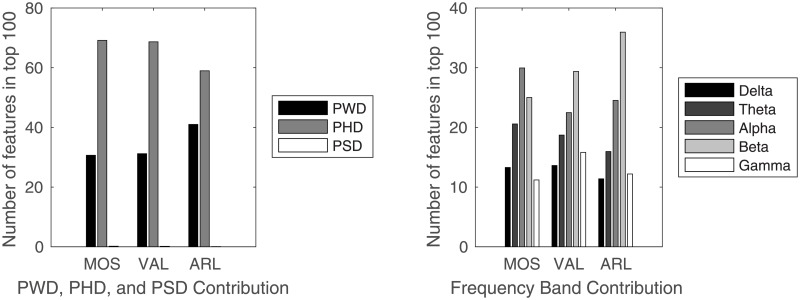
Contribution of features. Feature contributions were calculated by taking magnitude of their regression coefficients. (Left) Numbers of PWD, PHD, and PSD among the one hundred features that most greatly contributed to the prediction of each rating scale among all features. (Right) Number of features of each frequency band among the one hundred features that most greatly contributed to each rating scale.

**Fig 4:**
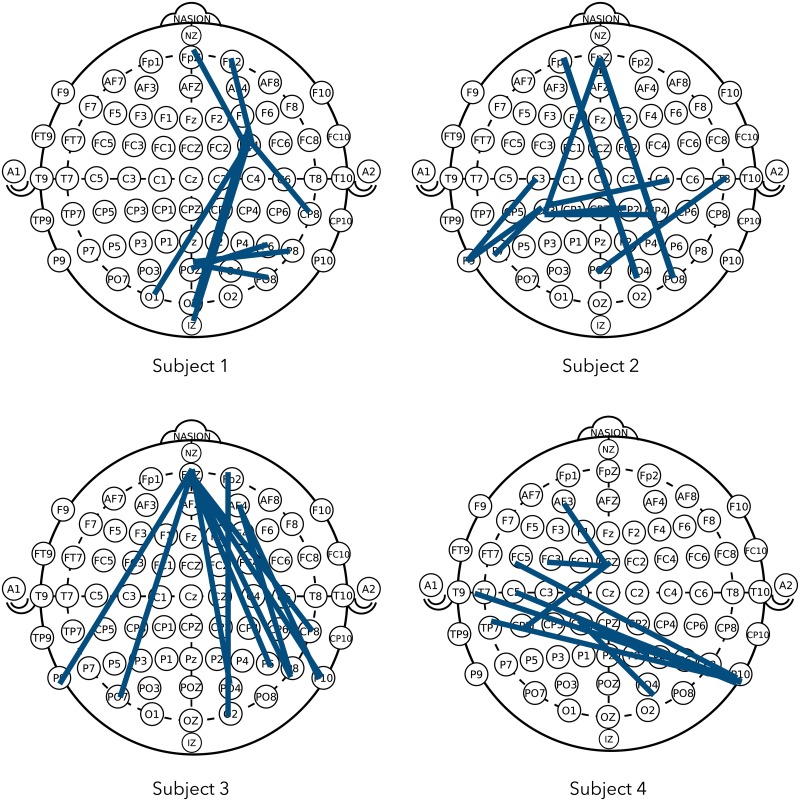
Contribution of channel pairs. Top ten channel pairs that contributed the most to MOS predictions of subjects 1, 2, 3, and 4. This figure was made by modifying the original one, which is distributed under the public domain dedication [44].

Table 2 summarizes RMSE, and the numbers of latent factors identified by PLS and HOPLS, respectively. Predictions with tensorial features generally made smaller errors than the vectorized ones for all the rating scales. Fig 3 reports the one hundred features that contributed to the prediction the most greatly, where feature contributions were calculated by taking the magnitude of the regression coefficients and PSD, PWD, and PHD are separately shown. PSD rarely appeared in the list of the top one hundred features list for all of the rating scales. Among the five frequency bands, the alpha band contributed the most to the MOS prediction, followed by the beta band. For the VAL and the ARL predictions, the beta band contributed the most, followed by the alpha band. The top ten channel pairs, which contributed the most to the MOS prediction extracted from subjects 1, 2, 3, and 4, are shown in Fig 4.

**Table 2:** Prediction results and the number of latent factors.

subject	RMSE (vector)			RMSE (tensor)			*R*_1_	*L*_1_	*L*_2_	*L*_3_	*R*_2_
	MOS	VAL	ARL	MOS	VAL	ARL					
1	1.091	1.090	1.086	0.961	0.957	0.962	1	3	4	5	3
2	1.042	1.057	1.058	0.987	0.958	0.975	1	4	4	4	3
3	0.997	1.018	1.020	0.907	0.936	0.931	2	7	3	4	2
4	1.148	1.110	1.043	0.949	0.944	0.994	2	7	5	2	2
5	1.187	1.225	1.229	1.082	1.177	1.150	1	6	7	4	5
6	1.260	1.292	1.151	1.000	2.176	1.941	4	7	4	4	4
7	0.979	0.981	1.007	0.869	0.935	1.167	1	2	3	5	5
8	1.155	1.186	1.125	0.970	0.989	1.017	1	1	5	1	1
9	1.215	1.221	1.160	0.996	0.994	1.023	2	7	7	1	2
10	1.111	1.112	1.022	0.957	0.985	1.144	1	5	4	3	4
11	1.125	1.243	1.0.19	1.013	1.047	0.920	3	7	7	1	1
12	0.996	1.193	1.177	0.641	0.912	0.680	29	4	2	3	4
13	1.258	1.227	1.234	1.051	1.050	1.035	3	4	2	5	4
14	0.991	1.102	1.040	0.940	0.980	0.929	12	7	1	2	3
15	1.022	0.989	0.969	0.965	0.927	0.934	1	7	3	2	5
16	1.196	1.206	1.087	1.058	1.047	1.027	4	7	6	2	5
17	1.021	1.083	1.087	0.884	0.886	0.924	3	4	7	4	3
18	1.055	1.027	1.092	0.915	0.920	0.969	2	1	3	4	4
19	1.130	1.142	1.126	1.021	1.081	1.020	1	5	1	5	4
20	0.995	1.028	0.944	0.887	0.990	1.057	1	4	5	2	5
21	1.121	1.157	1.103	0.900	0.969	0.997	1	1	1	4	2

## Discussion

Channel-pair-based features (PWD and PHD) contributed more to the predictions than channel-based ones (PSD), which agrees with a previous study [31] and suggests the importance of considering scalp EEG dynamics between brain regions, and that graph theory based features or functional connectivity analysis can be effective [45, 46]. The importance of spectral differences in caudality (DCAU) between the anterior and posterior [12, 47] or the front-posterior brain regions [31] as well as the lateral (left-right) spectral difference (DLAT) have been documented [28, 30]. In this study, both of DLAT and DCAU contributed to the predictions (Fig 4) although their effectiveness was dependent on the subjects.

Quality prediction models were independently trained for each subject in this study because emotion regulation is reportedly dependent on individuals [48]. The commonality of the channels/channel pairs, which greatly contributed to the predictions, was actually rather small (Fig 4). Therefore, creating subject-independent features is an interesting future work. However, note that the alpha and beta bands commonly contributed to the predictions, whereas the effective channels/channel pairs differed depending on the subjects. The alpha and beta bands contributed more largely to the predictions than the other frequency bands, which is in line with previous neurophysiological studies. The relationship between alpha band asymmetry and the withdrawal or disengagement from a stimulus or negative valence has been well documented in response to a variety of stimuli, including pictures [49, 50], music [31, 47, 51], movies [52], and speech [11, 13]. The beta band, which contributed the most to the ARL predictions, is reportedly associated with arousal and emotional experiences [53, 54].

Gupta et al. [13] predicted MOS values using the same data set that we used in this study. Their study used a simple linear regression model with not only EEG but also speech features. They reported the RMSE of their model was 0.117, which is much lower than our model, and suggests that speech features are much more informative than EEG features to predict subjective quality ratings.

Although we predicted the response values of MOS, VAL, and ARL, other perpetual dimensions were also proposed recently to model emotions or perceived quality-of-experiences [55, 56], which should be investigated in future research.

Neither previous work nor our current study advocate that physiological assessment methods of speech quality should replace subjective rating methods or signal analysis methods because, as stated in Introduction, each method has its own advantages and disadvantages and they can complement each other.

Several open questions remain. First, features were extracted and constructed as tensors as described in Feature Extraction and Regression Analysis, but other features and construction ways are also possible. For example, if time-frequency analysis is employed, times frames can be treated as one of the tensor modes. Second, this study analyzed the overall quality of each speech stimulus longer than ten seconds. However, parts of speech can affect much more largely its overall perceived quality. Therefore, analysis methods to specify such parts need to be studied.

## Conclusion

This study predicted the subjective quality ratings of synthesized speech solely based on EEG. We created vectorized and tensorial features for the regression that include channel-based and channel-pair-based features at multiple frequency bands. The experimental result showed that tensorial features more effectively predicted the subjective ratings than the other, and the trained predictive models were neurophysiologically plausible.
